# The role of cGMP as a mediator of lipolysis in bovine oocytes and its effects on embryo development and cryopreservation

**DOI:** 10.1371/journal.pone.0191023

**Published:** 2018-01-23

**Authors:** Kátia R. L. Schwarz, Fernanda C. de Castro, Letícia Schefer, Ramon C. Botigelli, Daniela M. Paschoal, Hugo Fernandes, Cláudia L. V. Leal

**Affiliations:** 1 Universidade de São Paulo (USP), Faculdade de Zootecnia e Engenharia de Alimentos (FZEA), Departamento de Medicina Veterinária, Pirassununga, São Paulo, Brasil; 2 Universidade Estadual Paulista (Unesp), Instituto de Biociências de Botucatu (IBB), Departamento de Farmacologia, Botucatu, São Paulo, Brasil; University of Central Florida, UNITED STATES

## Abstract

This study aimed to determine the influence of cyclic guanosine 3’5’-monophosphate (cGMP) and cGMP-dependent kinase (PKG) during *in vitro* maturation (IVM) on lipolysis-related parameters in bovine cumulus-oocyte complexes (COCs), and on embryo development and cryosurvival. COCs were matured with cGMP/PKG modulators and assessed for metaphase II rates (MII), cGMP levels, lipid content in oocytes (OO), transcript abundance for genes involved in lipolysis (ATGL) and lipid droplets (PLIN2) in cumulus cells (CC) and OO, and presence of phosphorylated (active) hormone sensitive lipase (HSL^ser563^) in OO. Embryo development, lipid contents and survival to vitrification were also assessed. Phosphodiesterase 5 inhibition (PDE5; cGMP-hydrolyzing enzyme) with 10^-5^M sildenafil (SDF) during 24 h IVM increased cGMP in COCs (56.9 vs 9.5 fMol/COC in untreated controls, p<0.05) and did not affect on maturation rate (84.3±6.4% MII). Fetal calf serum (FCS) in IVM medium decreased cGMP in COCs compared to bovine serum albumin (BSA) + SDF (19.6 vs 66.5 fMol/COC, respectively, p<0.05). FCS increased lipid content in OO (40.1 FI, p<0.05) compared to BSA (34.6 FI), while SDF decreased (29.8 and 29.6 FI, with BSA or FCS, respectively p<0.05). PKG inhibitor (KT5823) reversed this effect (38.9 FI, p<0.05). *ATGL* and *PLIN2* transcripts were detected in CC and OO, but were affected by cGMP and PKG only in CC. HSL^ser563^ was detected in OO matured with or without modulators. Reduced lipid content in embryos were observed only when SDF was added during IVM and IVC (27.6 FI) compared to its use in either or none of the culture periods (34.2 FI, p<0.05). Survival to vitrification was unaffected by SDF. In conclusion, cGMP and PKG are involved in lipolysis in OO and possibly in CC and embryos; serum negatively affects this pathway, contributing to lipid accumulation, and cGMP modulation may reduce lipid contents in oocytes and embryos, but without improving embryo cryotolerance.

## Introduction

Cellular lipolysis is mediated by catecholamines that raise the level of cyclic adenosine monophosphate (cAMP), which activates the cAMP-dependent protein kinase (PKA) that phosphorylates lipolysis-related proteins such as hormone sensitive lipase (HSL) and perilipins [1]. In adipocytes from humans and primates, the cyclic guanosine 3'5'-monophosphate (cGMP) pathway, which activates the cGMP-dependent protein kinase (PKG), will also phosphorylate proteins involved in the lipolytic process, including HSL and perilipin [2,3]. Therefore, lipolysis can also be mediated via cGMP / PKG and not only by the classical cAMP/PKA pathway [4, 5, 6]. Cyclic GMP is recognized as an important second messenger for extracellular signals such as nitric oxide (NO) and/or natriuretic peptides (NPs), which stimulate its synthesis by the enzyme guanylate cyclase (GC) [7]. Its physiological effect is determined by the activity of PKG [8] in response to its cellular level, which is regulated by the balance between its synthesis by GC and degradation by specific phosphodiesterases (PDE5, PDE6 and PDE9; [9]). PDE5 has been described in adipocytes and reported to participate in the regulation of the cGMP/PKG system [10]. PDE5 has been reported in oocytes and cumulus cells of mouse [11], swine [12] and cattle [13].

As reported in adipocytes, the elevation of cAMP levels by forskolin, stimulates lipolysis and reduces the amount of lipids in porcine oocytes and embryos [14, 15] and bovine oocytes [16]. In these studies, reduced lipids led to an improvement in cryotolerance. In a recent study, the increase in cGMP levels reduced the lipid content in bovine oocytes matured in the presence of fetal calf serum (FCS) [17]. However, its mechanism of action is still unknown, as well as its effect on cryotolerance.

The accumulation of cytoplasmic lipids observed in *in vitro* produced (IVP) embryos has been suggested as one of the causes for their reduced post-cryopreservation survival [18,19,20,21]. Oocytes matured in media enriched with FCS present higher amounts of cytoplasmic lipid droplets in relation to those matured *in vivo* [21]. One of the causes of this accumulation would be the uptake of lipids from the serum present in the medium [22]. However, a negative influence of FCS on cGMP levels was also observed [17]. Its presence during maturation decreased the relative expression of cGMP synthesis enzyme (GUCY1B3 isoform) and increased of the degradation enzyme (PDE5A isoform). In other words, the presence of FCS influences the transcription of genes that may be contributing to keep cGMP low throughout maturation, which could favor the accumulation of lipids [17].

Triglycerides (TAGs) are the most abundant lipids in oocytes and embryos [23]. The sequential hydrolysis of TAGs to their constituent molecules, one glycerol and three fatty acids, consists of cellular lipolysis, which occurs in basal or stimulated situations. Lipolysis results from the sequential activity of adipose triglyceride lipase (ATGL), HSL and monoacylglycerol lipase (MGL) [24]. ATGL, a member of a family of lipid metabolizing enzymes in mammals, is primarily located on the surface of the lipid droplet, and is responsible for the basal hydrolysis of TAGs to diacylglycerol (DAG) and a first fatty acid molecule. In the stimulated state, the phosphorylation of some perilipins (PLINs) and HSL by PKA and PKG generates an important modification on the lipid droplet (LD) surface, promoting the release of the ATGL coactivator and the translocation of HSL from the cytoplasm to the surface of the LD to promote lipolysis [25]. DAG is then converted to monoacylglycerol (MAG) and a second fatty acid is released by the action of HSL together with ATGL. Finally, MGL hydrolyzes DAG to monoacylglycerol (MAG), producing glycerol and a last free fatty acid [26]. PLIN1, PLIN2 and PLIN3 are members of the PAT family of proteins [27], which controls the interaction between lipases and LDs; indeed, PLIN2 has been shown to be associated with lipid accumulation [28]. PLIN2 has been identified in mouse [29], porcine [30] and bovine oocytes [31] and bovine embryos [32]. In these studies, PLIN2 was shown to co-localize with LDs, indicating its role as a major LD protein in these cells.

The objective of this work was to determine the interference of serum and cGMP during IVM on lipid metabolism in bovine oocytes and its consequences on the development and cryopreservation of bovine embryos. SDF, a potent and selective inhibitor of PDE5 [33], was used to reduce cGMP hydrolysis in order to maintain its intracellular levels for the activation of its dependent pathways. With our findings, knowledge about the complex regulation of lipolysis in COCs, influenced by cGMP, has been further expanded.

## Materials and methods

### Media and chemicals

Chemicals were purchased from Sigma Chemical (St Louis, MO, USA), unless otherwise stated. All stock solutions were stored at -20°C until the day of the experiment.

### Cumulus-oocyte complexes collection

Bovine ovaries (*Bos taurus*) were collected from commercial slaughterhouses in Ipuã/SP (“Olhos D’agua”—1420 Regina Mosconi Avenue, Zip Code 14.610–000 (-20.444.241, -48.013096)) and Sertãozinho/SP (“Barra Mansa”—10 Atílio Balbo Street (-21.161314,—47.955342)); ovaries were from cross-bred cows with varying levels of blood mix from *Bos taurus taurus* and *Bos taurus indicus* animals. Immediately after slaughter ovaries were transported in sterile saline solution with antibiotics (100 IU/mL penicillin and 100 mg/mL streptomycin) at 30°C. In the laboratory, 2 to 8 mm follicles were aspirated with an 18 "G" needle attached to a disposable 10 mL syringe. The aspirated follicular fluid was placed in 50 mL conical tubes and maintained for 5 min for sedimentation. The upper portion of the liquid was removed and the remaining portion was added with 3 to 5 mL washing medium [TCM199 with 25 mM Hepes, 10 μg/mL gentamycin and 1% fetal calf serum (FCS, Gibco-BRL, Grand Island, NY, USA). The material was then transferred to a Petri dish (60 x 15 mm) under a stereomicroscope for selection of grade I and II cumulus-oocyte complexes (COCs) [34].

### *In vitro* maturation

For *in vitro* maturation (IVM), the selected bovine COCs were cultured in maturation medium [TCM199 with 20 mM bicarbonate containing 0.2 mM sodium pyruvate, 0.5 μg/mL follicle stimulating hormone (FSH), 5.0 μg/mL luteinizing hormone (LH) and 10 μg/mL gentamicin] with 0.4% bovine serum albumin (BSA) or 10% FCS. Groups of 20 COCs were randomly distributed into droplets of each treatment (100μL maturation medium under mineral oil) and incubated at 38.5°C and 5% CO_2_ in air and maximum humidity for 24 h.

### Determination of nuclear maturation rate

After maturation, cumulus cells (CC) were removed from COCs in 200 μL PBS+PVA by vortexing in disposable plastic microtubes for 4 min. The denuded oocytes (DO) were washed 3x in PBS+PVA and stained with Hoechst 33342 (10 μg/mL in PBS+PVA) for 15 min protected from light. After washing 3x in PBS+PVA, the samples were transferred to a glass slide (~12 oocytes/slide) containing 5 μL Pro Long Gold (Invitrogen), which was carefully covered with a coverslip. The analysis was done under an epifluorescence microscope (Nikon Eclipse—filters with 475-490nm excitation and 445-450nm emission) to determine the maturation rate (oocytes were considered as matured when reaching metaphase II stage, when chromatin was seen as a metaphase plate and the first polar body was present).

### Cyclic GMP measurements

Cyclic GMP levels were measured by the enzyme immunoassay method (EIA) in pools of 40 COCs. Great care was taken to select COCs with similar size of cumulus investments [35]. COCs were washed three times in droplets of TCM199 with 20 mM Hepes and 0.4% BSA and transferred to 200 μL 0.1 N HCl for 20 min to lyse the cells. After this period, the samples were centrifuged at 12.000 g for 5 min. The supernatant was transferred to new tubes and stored at -20°C until analyzed by immunoassay according to instructions of the kit (Direct Cyclic GMP EIA ^®^ Enzo Life Sciences, Ann Arbor, MI, USA).

### Determination of lipid content with the fluorescent probe Nile Red

For oocyte staining, cumulus cells were removed from COCs using 0.3 IU hyaluronidase in PBS+PVA. DOs or embryos were washed in PBS+PVA, fixed in 4% paraformaldehyde (PFA) and simultaneously permeabilized with 0.5% Triton X-100 solution in PBS+PVA for 15 min at room temperature and then washed again 3x in PBS+PVA. Oocytes/embryos were stained with 1 μg/mL Nile Red in PBS+PVA for 30 min, protected from light and at room temperature. After this period, the oocytes/embryos were washed 3x in PBS+PVA and transferred to a glass slide (12 oocytes or 6 embryos/slide) containing 5μL Pro Long Gold (Invitrogen), which was carefully covered with a coverslip. The fluorescence of Nile Red was evaluated under an epifluorescence microscope (Nikon Eclipse- TS100/filter G2A; excitation 515–560 nm and emission greater than 590 nm) with fast resolution [36]. The photographs of stained oocytes/embryos (20x magnification) were taken at 80 ms exposure and the fluorescence intensity (FI) was measured using ImageJ software. The oocytes/embryos lipid contents are presented as mean fluorescence intensity (FI) [15].

### Quantitative real time polymerase chain reaction (qPCR)

Groups of 20 COCs were denuded by pipetting in PBS+PVA. The partially DOs were removed from the solution and vortexed for 4 min in PBS+PVA to remove any remaining CC. After washing in PBS+PVA, DO pools were stored at -80°C in 1 μL PBS+PVA with 1.0 U/μL RNase OUT (Invitrogen, Carlsbad, CA, USA). The solution containing the CC was centrifuged at 900 g for 10 min. The supernatant was removed and the cell pellet treated with 1.0 U/μL RNase OUT and stored at -80°C until use. All samples of CC and DO collected after IVM (five replicates) were submitted to total RNA extraction according to the Trizol^®^ protocol (Invitrogen TM). After extraction, total RNA samples were quantified by spectrophotometry using a NanoDrop^®^ 2000 (Thermo Scientific) to verify integrity assessing the 260/280 ratio (between 1.8 and 2.0). For DNA digestion and reverse transcription procedures CC and DO samples had their volumes adjusted to contain 1,000 ng RNA per sample. All samples were submitted to DNA digestion procedure using the enzyme DNAse I—Amplification Grade^®^ (Invitrogen). For reverse transcription (RT) and synthesis of complementary DNA (cDNA) of samples, the “High Capacity cDNA Reverse Transcription” kit was used (Applied Biosystems Carlsbad, CA-USA). Relative quantification of transcribed genes was performed by real time qPCR, using the SybrGreen^®^ detection system with Power SybrGreen^®^ PCR Master Mix reagent (Applied Biosystems TM), in the Applied Biosystems Step One equipment. qPCR reactions were run in 12 μL containing 0.25 mM of each primer, 1x SYBR Green PCR Master Mix (Applied Biosystems), 2.5 μL H_2_O and 2.0 μL template (two-fold diluted cDNA; 50ng). Cycling conditions for amplification were: 95°C for 10 min followed by 45 cycles at 95°C for 15 sec, 57°C for 20 sec and 60°C for 40 sec. Each sample was analyzed in duplicate for each of the genes. As negative control for the reaction, ultrapure DNAse and RNAse free water was used in place of cDNA. Values of expression of target genes *ATGL* and *PLIN2* were normalized by the geometric mean of mRNA transcripts of two housekeeping genes, *GAPDH* and *ACTB* [37]. Differences in frequencies of transcripts were calculated by the 2^-ΔΔCt^ comparative method [38]. A “threshold” line was fixed at the mean point of the exponential amplification to each gene. Primers were designed (Table 1), based on the bovine sequences available on the genome browser of the University of California Santa Cruz (UCSC) (http://genome.ucsc.edu, accessed December 2013), using the program Primer3 (Primer3web, version 4.0.0: http://primer3.ut.ee). All primers were tested for efficiency and the obtained amplicons were sequenced to ensure their specificity.

**Table 1:** Nuclear maturation rate in oocytes submitted to IVM with different concentrations of SDF for different periods of culture (0–24 h or 12-24h).

Sildenafil concentration	Oocytes N*	Period of culture with sildenafil	
		(0 to 24 h) N**(% MII ± SEM)	(12 to 24 h) N**(%MII ± SEM)
0 M (control)	160	80 (66.3±3.9) ^a^	80 (66.3±3.9)
10^−7^ M	111	62 (74.2±1.5) ^ab^	49 (61.9±3.8)
10^−5^ M	114	51 (84.3±6.4) ^b^	63 (60.3±2.4)
10^−3^ M	155	76 (53.9±2.4) ^c^	79 (60.7±1.7)

*Includes total oocytes for the two periods of drug action;

** total oocytes per period.

Data are expressed as n (% mean ± SEM) of four replicates. Values with different letters in the same column show significant differences (p<0.05).

### Western blotting

Groups of 50 DOs were lysed in RIPA buffer (150 mM NaCl, 1% NP-40, 0.5% sodium deoxycholate, 0.1% SDS and 50 mM Tris, pH 8.0) containing protease and phosphatase inhibitors. The lysates were then centrifuged for 10 min, after which the supernatants were collected. Proteins were separated by electrophoresis on a 12% SDS polyacrylamide gel and blotted onto a polyvinylidene fluoride membrane (Millipore, Jeffrey, NH, USA). The membrane was blocked in SuperBlock^™^ T20 (TBS) Blocking Buffer (Thermo Scientific) for 1 h. After a brief wash in TBS-T, the membrane was incubated overnight at 4°C, with the primary antibody against phospho-HSL^ser563^ (1:200 dilution, #4139, Cell Signalling Technology) and β-Actin (monoclonal Anti-β-Actin-Peroxidase, 1:1000 dilution, A3854) diluted in tris-buffered saline (TBS) with 0.1% (v/v) Tween-20 (TBS-T) with 5% BSA. After three 15 min washes in TBS, the membrane was incubated in the secondary antibody anti-rabbit IgG HRP-linked (1:3000 dilution, #7074, Cell Signaling Technology) diluted in TBS-T for 1:30 h at room temperature. The membranes were again washed thrice in 1x TBS-T for 15 min. For detection, the membranes were subjected to a chemiluminescent reaction using the reagent ECL Plus Prime Western Blotting Detection System solution (Amersham^™^, Buckinghamshire, UK). Imaging and band density analyses were performed using ChemiDoc MP Image System (Bio-Rad,) and the software Image Lab 5.1, respectively.

### *In vitro* fertilization and embryo culture

For embryo production *in vitro*, matured COCs were transferred to 90 μL droplets (20 COCs per droplet) of fertilization medium covered with mineral oil. COCs were subjected to *in vitro* fertilization (IVF) using frozen semen (Nelore bull Ópio—CRV Lagoa, Sertãozinho, Brazil) of the same bull and of proven fertility. Spermatozoa were selected using the Percoll method, and the concentration was adjusted to 2 × 10^6^ sperm cells/mL. Fertilization was performed in droplets under mineral oil of TALP medium [39] supplemented with 5 mg/mL BSA (96% fatty acid-free), 0.2 mM/mL pyruvate, 30 μg/mL heparin, 18 μM/mL penicillamine, 10 μM/mL hypotaurine, 1.8 μM/mL epinephrine, 100 μg/mL streptomycin sulfate and 100 IU/mL penicillin (Gibco). COCs and spermatozoa were co-incubated under the same conditions (temperature, humidity and gas atmosphere) as for the *in vitro* maturation for 20 h. The day of fertilization was defined as Day 0 (D0). Presumptive zygotes were denuded by repeated pipetting and transferred into 90 μL droplets of culture medium under mineral oil (20 zygotes per droplet), and cultured under the same conditions as for *in vitro* maturation. The culture medium was synthetic oviduct fluid with amino acids (SOFaa) [40] supplemented with 2.7 mM/mL myo-inositol, 0.2 mM/mL pyruvate, 2.0% fetal calf serum (FCS; v/v), 5 mg/mL BSA (fatty acid-free), 100 μg/mL streptomycin sulfate and 100 IU/mL penicillin (Gibco). After 96 h (D4) of culture, cleavage was checked and non cleaved embryos were discarded. The embryos remained in culture until Day 7 (D7). Blastocyst production and lipid content were evaluated on D7. Blastocyst rates were calculated relative to total COCs matured in each treatment.

### Vitrification and warming of embryos

Vitrification was performed with blastocysts (Bl) and expanded blastocysts (Xb). The protocol of Campos-Chillon [41] was followed with some modifications. The embryos were exposed to vitrification medium 1 (VM1) composed of 5 M ethylene glycol (EG) in PBS with 0.1% PVA, 0.4% BSA, 0.2 mM/mL pyruvate and 10 μg/mL gentamicin (Base Medium—BM) for 3 min and transferred to a 5 μL droplet of vitrification medium 2 (VM2) composed of 7 M EG, 0.5 M galactose (GL) and 18% ficoll 70 in BM. Immediately afterwards, a 0.25 mL straw (Nutricell^®^) was prepared containing 1 cm GL (1 M in BM), 0.5 cm air, 7 cm GL, 0.5 cm air, a 5 μL droplet of VM2 containing the embryos, 0.5 cm air and then GL until the straw was full. Each straw contained two to four embryos. The straws remained for 1 min in N_2_liquid vapor before immersion into the liquid. For warming, straws were kept in air for 8 s and then in water at 37°C for 15 s. The embryos were then washed and cultured in SOFaa containing 10% FCS and incubated at 38.5°C and 5% CO_2_ in air and maximum humidity for 12 h to assess embryo re-expansion and for 48 up to 72 h to assess embryo hatching.

### Statistical analysis

Statistical analyses were performed using the SAS System (V 5.1; SAS Institute, Inc., Cary, NC), by one-way ANOVA followed by Tukey post hoc test. Data from five replicates/experiment were tested for normal distribution and homogeneity of variance and were transformed to arcsine (embryonic development) or log10 (measurement of cGMP and lipid content) when these criteria were not met. For gene expression the values of 2^-ΔΔct^ were considered [38]. All means are presented ± standard error of the mean (S.E.M.). Differences with probabilities of p<0.05 were considered significant.

## Results

### Effect of different concentrations of the PDE5 inhibitor (SDF) on nuclear maturation rate

To determine culture conditions with the PDE5 inhibitor during IVM, different concentrations of SDF (10^−7^, 10^−5^ and 10^-3^M) were added to the maturation medium consisting of TCM199 supplemented with 0.4% BSA. As cGMP levels can affect nuclear maturation control, maturation rate was assessed to certify treatments to induce lipolysis would not have adverse effects on maturation. Therefore, after 24 h IVM with different treatments, COCs were evaluated for the nuclear maturation rate. When SDF was added from the beginning of maturation (0-24h), the highest concentration of the PDE5 inhibitor (10^-3^M SDF) reduced maturation rate (53.9±2.4%, p<0.05) relative to the other concentrations (74.2±1.5 and 84.3±6.4% MII for 10^-7^and 10^−5^ M SDF, respectively) and in relation to the untreated control (66.3±3.9%). On the other hand, the intermediate concentration (10^−5^ M SDF) seems to have favored nuclear maturation, increasing the number of oocytes that concluded meiosis (84.3±6.4% MII; p<0.05) relative to controls. When SDF was added only on the second half of IVM (12–24 h), there was no interference on maturation rate which was around 62.4% MII for all concentrations tested and control (p>0.05, Table 1).

As the highest concentration of SDF throughout IVM reduced maturation rates, this group was excluded from the next experiment.

### Effect of inhibition of PDE5 with SDF on cGMP levels in COCs matured *in vitro*

**Fig 1:**
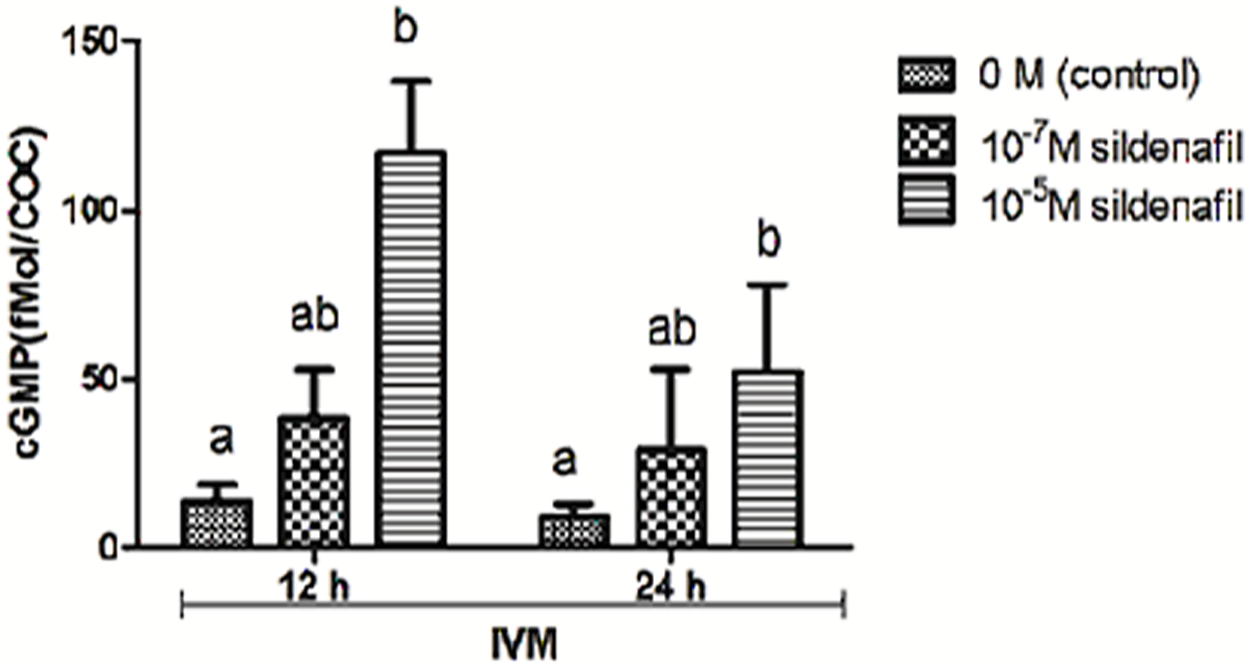
cGMP levels in bovine COCs matured *in vitro* for 12 and 24 h in the presence of different concentrations of the PDE5 inhibitor (0, 10^−7^ and 10^−5^ M SDF) in TCM199 supplemented with 0.4% BSA (control). Data are the mean ± SEM of four replicates. Different letters indicate significant differences (p<0.05).

To determine the effects of SDF treatments on cGMP levels, COCs were matured for 12 and 24 h in TCM 199 supplemented with 0.4% BSA. Inhibition of PDE5 by 10^−5^ M SDF for 12 h (115.2±35.5 fMol/ COC) and 24 h IVM (56.9±14.8 fmol/COC, p<0.05, Fig 1) increased cGMP levels relative to untreated controls (p<0.05) at the same time points (12.3±1.6 and 9.5±2.2 fMol/COC, for 12 and 24 h, respectively).

These results confirmed the effect of SDF on inhibiting PDE5, which in turn resulted in increased cGMP levels during the periods studied. As the 10^-5^M SDF concentration increased cGMP levels in relation to the control group, it was selected for the next experiments.

### Effect of fetal bovine serum and inhibition of PDE5 with SDF on the level of cGMP in COCs matured *in vitro*

**Fig 2:**
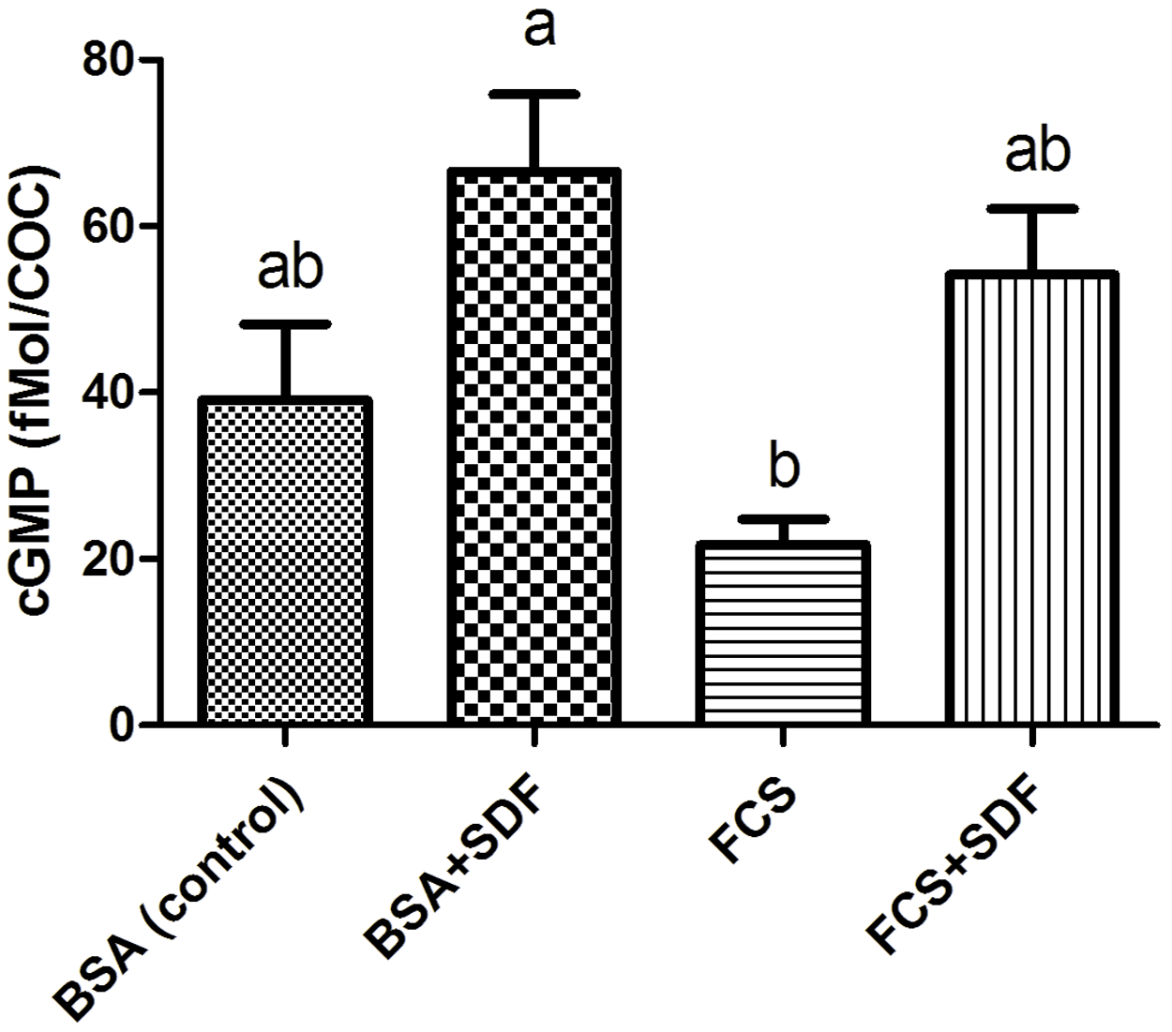
cGMP levels in bovine COCs matured *in vitro* for 24 h in in the presence or not of the PDE5 inhibitor (10^−5^ M SDF) in TCM199 supplemented with 0.4% BSA or 10% FCS. The control group consists of COCs matured with 0.4% BSA without addition of FCS or SDF. Data are the mean ± SEM of four replicates. Different letters indicate significant differences (p<0.05).

To determine the effect of serum and inhibition of PDE5 on cGMP levels, COCs were matured for 24 h in TCM199 medium supplemented with 0.4% BSA or 10% FCS, with or without 10^−5^ M SDF. The presence of FCS resulted in reduced cGMP levels compared to the BSA group with SDF (19.6±3.2 vs 66.5±9.3 fMol/COC, respectively, p<0.05). The addition of SDF to the medium supplemented with BSA or FCS (54.2±7.91 fMol/COC) resulted in higher levels of cGMP, but did not differ in relation to the control (BSA only, 39.1±9.1 fMol/COC, p>0.05, Fig 2). Thus, serum appears to interfere with cGMP levels and inhibition of PDE5 with SDF, particularly in the presence of BSA, can restore cGMP levels.

### Effect of serum and inhibition of PDE5 with SDF on lipid content of oocytes matured *in vitro*

**Fig 3:**
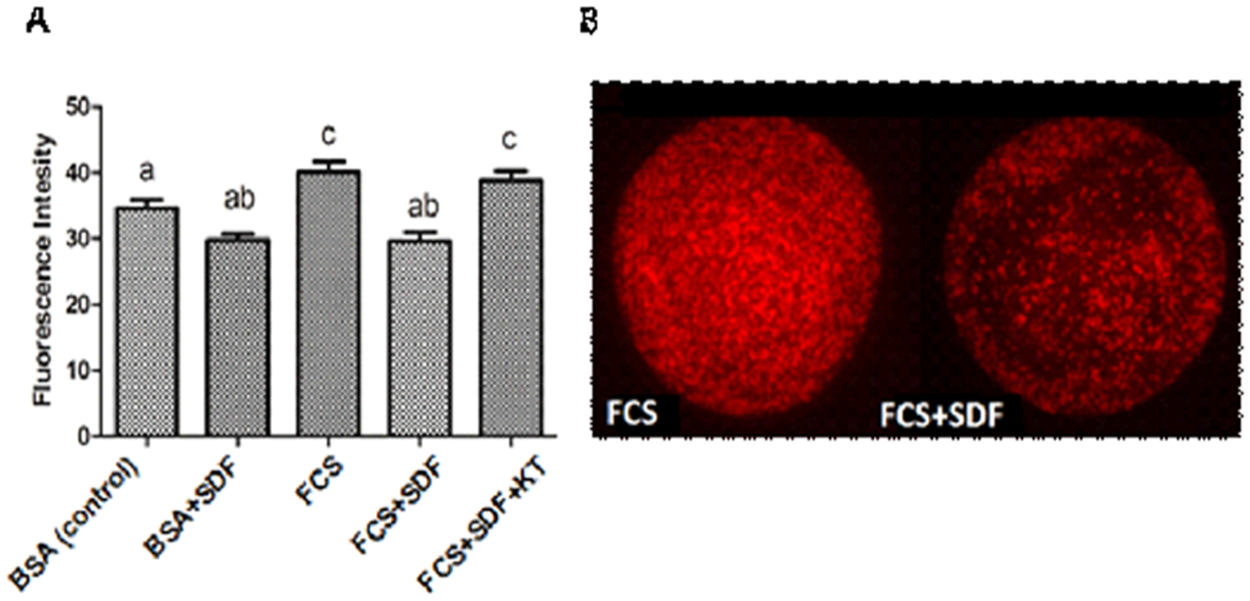
Total amount of lipids in bovine oocytes matured *in vitro* for 24 h. A) Lipid content in oocytes with different protein sources (0.4% BSA or 10% FCS) with or without phosphodiesterase 5 inhibitor (10^−5^ M SDF) associated or not with PKG inhibitor (10^-5^M KT5823). B) Representative image (20x magnification) of Nile Red stained FCS and FCS + SDF treated oocytes. The control group consists of COCs matured with 0.4% BSA without addition of FCS or SDF. Data are expressed as the mean ± SEM of six replicates. Values with different superscript letters differ significantly (p<0.05). FI, fluorescence intensity (arbitrary units).

Inhibition of PDE5 with 10^−5^ M SDF did not significantly reduce lipid content in oocytes matured with BSA (29.8±0.9 FI; p>0.05) in relation to the control with BSA only (34.6±1.2 FI, Fig 3). However, in oocytes matured in the presence of FCS with SDF, there was a reduction in lipid content (29.6±1.3 FI, p<0.05) compared to the group matured only with FCS without the addition of the PDE5 inhibitor (40.1±1.6 FI). Oocytes matured only with FCS presented the highest lipid content (p<0.05) in relation to the other treatments. When COCs were matured with FCS and SDF associated with the PKG inhibitor (10^−5^ M KT5823), there was a reversal of the effect of SDF with an increase in lipid amounts (38.9±1.4 FI; p>0.05, Fig 3) at levels similar to those of COCs matured only with FCS.

Thus, serum seems to interfere negatively on the lipolytic pathway in oocytes inducing lipid accumulation. The addition of SDF reduced lipid content, indicating that the effect of serum is given, at least in part, through cGMP levels. Furthermore, the lipid lowering effect given by cGMP elevation appears to be related to PKG activation by the nucleotide, since the inhibition of this protein kinase was able to reverse the effect of SDF. Therefore, the cGMP-PKG pathway may participate on the regulation of lipolytic activities in the oocyte.

### Effect of serum and inhibition of PDE5 with SDF on transcripts related to lipid metabolism

**Fig 4:**
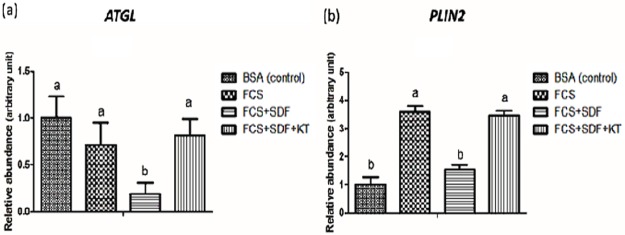
Relative abundance of transcripts for genes related to lipid metabolism in cumulus cells after 24 h IVM with BSA (control) or 10% FCS with or without phosphodiesterase 5 inhibitor (10^−5^ M SDF) associated or not with PKG inhibitor (10^−5^ M KT). Data are expressed as the mean ± SEM of five replicates. (A) ATGL, adipose triglyceride lipase; (B) PLIN2, perilipin 2.

The interference of serum and the cGMP pathway was evaluated on the expression of genes involved in lipolysis (*ATGL*) and lipid accumulation (*PLIN2*) in cumulus cells and oocytes from COCs matured for 24 h. Both transcripts were detected in both cell types. In CC, the relative expression of *ATGL* was negatively influenced by the inhibition of PDE5. Its expression was reduced in relation to control (BSA) and treatment with FCS (p<0.05). The association of SDF with the PKG inhibitor (KT5823), however, restored the level of transcripts similar to the BSA and FCS groups (p<0.05, Fig 4). These data suggest that although *ATGL* is not affected by serum, SDF inhibits its transcription by elevating cGMP, indicating nucleotide influence on its expression. In addition, the effect of cGMP increase appears to be given by PKG activation since its inhibition in the presence of SDF reversed such effect.

*PLIN2*, considered to be an indicator of lipid accumulation, had greater expression in cumulus cells from COCs cultured with FCS compared to the control group (BSA, p<0.05), which may indicate an accumulation of lipids in these cells. Inhibition of PDE5 reversed this effect by reducing the expression of *PLIN2* relative to the FCS treated group in the absence of its inhibitor (p<0.05). Thus, there appears to be a relationship between FCS increasing *PLIN2* transcripts and SDF, via elevated cGMP, to cause their reduction. In addition, the association of the PKG inhibitor KT5823 resulted in increased expression of this transcript relative to the SDF treated group (p<0.05; Fig 4b), suggesting that the effect of SDF is probably mediated by PKG.

The genes previously studied in cumulus cells had their relative expressions identified in oocytes, but none of the treatments had a significant effect (p>0.05). This observation suggests that cumulus cells are more sensitive to changes in their lipid metabolism and that such changes may have effects on oocyte lipids.

### Detection of phosphorylated HSL in oocytes matured *in vitro* with cGMP modulators

**Fig 5:**
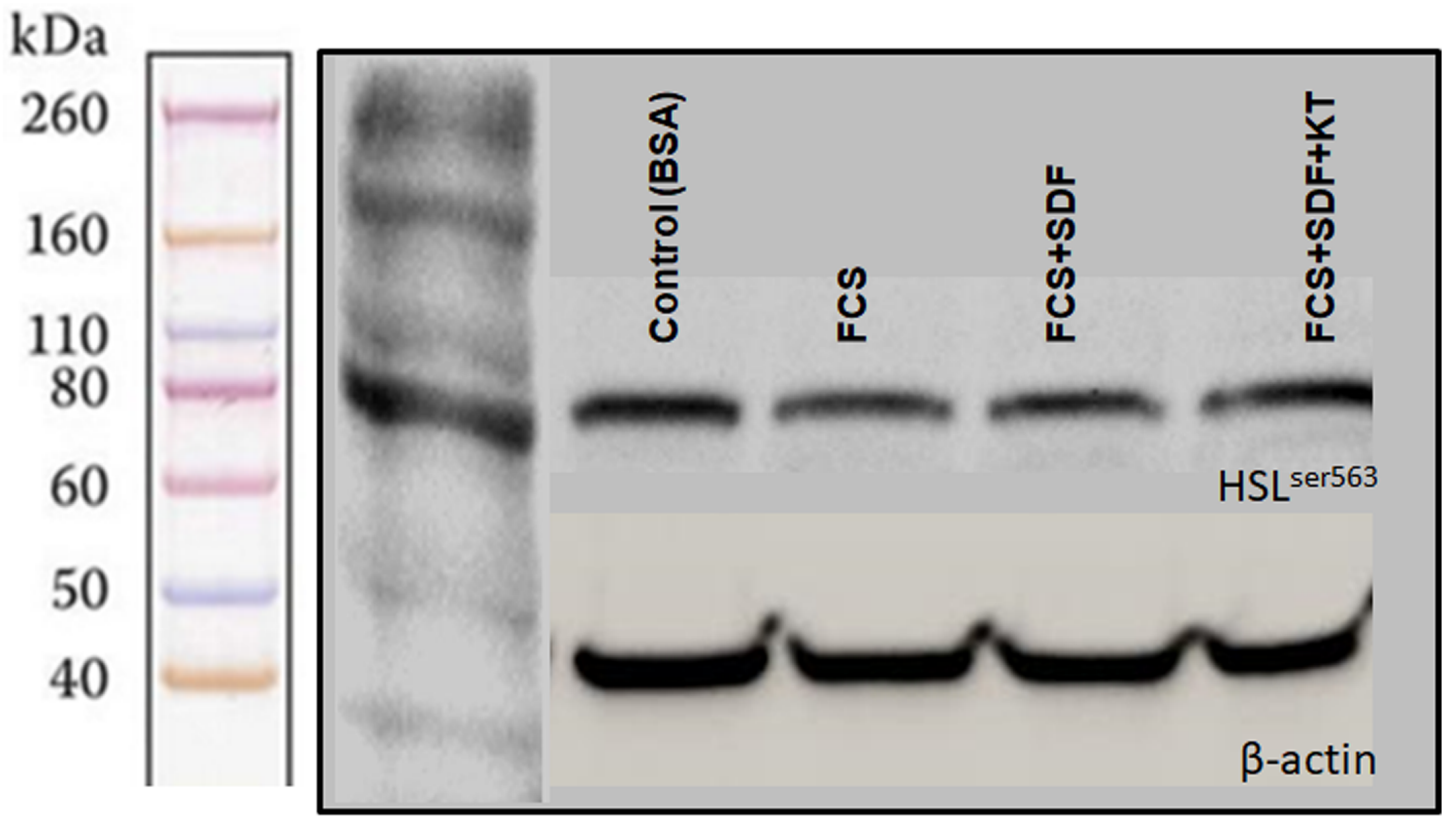
Immunodetection of HSL^ser563^ by western blotting in oocytes after 24 h of IVM with 10^−5^ M sildenafil (PDE5 inhibitor) associated or not with 10^−5^ M KT5823 (PKG inhibitor). The control group was matured in TCM199 + 0.4% BSA and beta-actin was used as internal control. A pool of 50 DOs was used to extract the protein. Data are expressed as the mean ± SEM of four replicates. Significant differences represented by different letters (p<0.05).

The HSL protein phosphorylated on serine 563 (active form) was detected in bovine oocytes originating from COCs matured in the presence of modulators of the cGMP pathway, indicating lipolytic activity during maturation in all groups evaluated (Fig 5).

### Effect of PDE5 inhibition during *in vitro* maturation and / or culture on embryonic development and on the lipid content of embryos produced *in vitro* in the presence of FCS

Interference of the cGMP pathway on embryo development was tested indifferent periods of *in vitro* culture, in which SDF was added during IVM, IVC, both periods or not added at all (control). Inhibition of PDE5 during any or both culture periods did not interfere with cleavage and blastocyst rates compared to untreated controls, which were around 84.6% and 43.0%, respectively, for all treatments (p>0.05, Table 2).

**Table 2:** Development of *in vitro* produced embryos cultured in the presence of FCS associated or not with SDF during IVM and/or IVC culture.

Treatments	Oocytes (N)	Cleavage (%)*	Blastocysts (%)**
IVM/IVC (Control)	308	79.6 ± 2.6	42.9 ± 4.7
IVM+/IVC	186	88.8 ± 3.6	41.4 ± 1.2
IVM/IVC+	187	86.7 ± 3.8	41.1 ± 0.3
IVM+/IVC+	239	83.4 ± 4.3	46.8± 2.9

* Cleavage rate after 96h IVF (D4) calculated relative to the total number of oocytes.

** Blastocyst rate 7 days after IVF (D7) calculated relative to the total number of oocytes.

(+) SDF = 10^−5^ M SDF.

Control group was cultured in the absence of drugs in the IVM and IVC periods. Data are expressed as the % mean ± SEM of five replicates. (p>0.05).

In order to evaluate the interference of PDE5 inhibition in different steps of *in vitro* culture on the lipid content of the embryos produced, D7 blastocysts were stained with the fluorescent probe Nile Red. When COCs were matured in the presence of FCS+SDF, the blastocysts produced showed higher amount of lipids (37.0±0.9 FI, p<0.05) in relation to the control (31.7±0.7 FI). When SDF was added only during IVC, embryos had unchanged lipid contents, which were similar to untreated controls (33.9±1.5 FI, p>0.05). Only when SDF was added during both culture periods (IVM and IVC), a reduction in lipid contents was observed (27.6±0.8 FI, p<0.05, Table 3). According to the previous results, the presence of SDF in the culture medium results in oocytes with lower lipid content, but these oocytes after being fertilized and cultured in the presence of serum, do not originate embryos with similar characteristics. The presence of SDF appears to act positively on lipolysis when it remains present for a longer period of time during both phases of culture.

**Table 3:** Lipid content of *in vitro* produced embryos cultured in the presence of FCS associated or not with SSDF during IVM and/or IVC culture.

Treatments	Blastocysts (N)	Lipid content* (FI)
IVM/IVC (Control)	132	31.7±0.7^a^
IVM+/IVC	138	37.0±0.9^b^
IVM/IVC+	77	33.9±1.5^ab^
IVM+/IVC+	100	27.6±0.8^c^

*Total amount of lipids in arbitrary units of fluorescence intensity (FI) in bovine blastocysts (D7) [15].

The control group corresponds to COCs matured in medium supplemented with 10% FCS and embryos cultured with 2% FCS. (+) SDF = 10^−5^ M SDF. Data are expressed as mean ± SEM of six replicates. Values with different letters in the same column show significant differences (p<0.05).

### Effect of PDE5 inhibition during *in vitro* maturation and / or culture on re-expansion and hatching after vitrification of embryos produced *in vitro* in the presence of FCS

Embryos from the groups in the previous experiment were evaluated regarding their cryotolerance to assess whether reduced lipid contents would improve embryo resistance to cryopreservation. When SDF was included during maturation or during both culture periods there seemed to be an increase in the production of embryos of higher quality for vitrification, since the proportion of embryos classified as grade 1 and 2 for was higher. In these groups it was possible to vitrify about 64% of the total blastocyst production, a proportion which was higher than in the groups that only had SDF supplementation during the IVC (49.5±4.3%) or the untreated controls (45.4± 3.5%, p<0.05). However, there was no influence of SDF on post-vitrification embryonic development, with re-expansion and hatching rates of 92 and 64%, respectively, for all groups (p>0.05, Table 4).

**Table 4:** *In vitro* survival (re-expansion) and hatching rates of vitrified bovine embryos produced *in vitro* with PDE5 inhibitor during IVM and/or IVC.

Treatments	Oocytes (n)	Blastocyst * (D7)	n (% ± SEM)		
			Embryos Vitrified**	Re-expansion (24h)***	Hatching (48-72h)***
IVM / IVC	330	92 (28.5 ± 3.4)	42 (45.4 ± 3.5)^b^	39 (92.7 ± 3.8)	26 (61.1 ± 4.3)
IVM+ / IVC	322	75 (23.3 ± 1.6)	48 (63.9 ± 3.8)^a^	46 (95.0 ± 5.0)	35(72.5 ± 11.4)
IVM / IVC+	336	117 (34.8 ± 2.0)	58(49.5 ± 4.3)^b^	53 (90.8 ± 4.7)	41 (70.0 ± 3.0)
IVM+ / IVC+	322	93 (28.8 ± 6.9)	59(63.5 ± 2.5)^a^	54 (91.5 ± 5.2)	32 (54.5 ± 3.5)

*Blastocyst production rates were estimated considering the total number of oocytes (IVF = day zero).

**Vitrified Embryos rates were estimated considering the total number of blastocysts.

***Re-expansion and hatching rates were estimated considering the total of vitrified embryos.

The control group corresponds to COCs matured in medium supplemented with 10% FCS and embryos cultured with 2% FCS. + = 10^-5^M SDF. Data are expressed as n (% mean ± SE) of four replicates. Values with different letters in the same column show significant differences (p<0.05).

Results suggest that treatment with SDF during both IVM and IVC reduces lipid contents in resulting embryos and increases proportion of better quality embryos for vitrification, but this effect does not translate into more cryotolerant embryos.

## Discussion

The *in vitro* production of embryos is the basis for the treatment of infertility in humans and is widely used in cattle to obtain a higher number of offspring from high genetic merit animals [42]. Although commercially successful, one important obstacle to further disseminate this technique is the sensitivity of such embryos to cryopreservation. The accumulation of cytoplasmic lipids observed in the IVP embryos has been pointed out as one of the causes for their reduced cryotolerance [18,19,21]. In the present study, manipulation of the cGMP pathway using the PDE5 inhibitor SDF has been investigated as an alternative to interfere on the lipolytic activities of oocytes and bovine IVP embryos. Also, the influence of serum on cGMP pathway and lipid contents was investigated.

The initial experiments were performed to determine adequate culture conditions to use SDF, in order to reduce cGMP hydrolysis by PDE5 and sustain nucleotide levels during culture (Fig 1), but without affecting oocyte nuclear maturation. Since cGMP is also involved in the resumption of meiosis [43, 44], different concentrations of the PDE5 inhibitor were tested, either during the entire maturation period or only after the meiosis resumption phase. When SDF was used after meiosis resumption, no effects were observed, but when it was added throughout the entire IVM period the highest concentration (10^−3^ M) reduced MII rates. These results (Table 1) confirm the influence of cGMP on the control of meiosis resumption as reported by Schwarz [12] in bovine oocytes and also in rodents [43, 44]. However, no effects were observed when a lower concentration was used (10^−7^ M) and maturation rates even increased with an intermediate concentration (10^−5^ M). Therefore, cGMP effects on nuclear maturation are dependent on concentration and period of exposure during IVM.

To confirm SDF treatment was not interfering with maturation, but was in fact sustaining cGMP levels, nucleotide levels were measured, and increased cGMP up to the end of maturation was observed using 10^−5^ M SDF. Therefore, the decrease in this nucleotide, which occurs during the maturation process [45], was avoided by SDF.

Thus, in experiments 1 and 2, it was possible to determine culture conditions with SDF, effective to elevate cGMP levels during IVM, without interfering with oocyte maturation. In the next experiments, we evaluated the effects of FCS on cGMP signaling pathway and its consequences on the lipid metabolism of bovine COCs.

FCS has been implicated in the increase in lipid content in embryos [23], with consequent reduction in embryo quality [46, 47] and lower cryotolerance [48, 49]. As the cGMP pathway can affect lipolysis in adipocytes [3], we evaluated effects of serum on this pathway related the accumulation of lipids in oocytes and embryos. COCs matured in the presence of FCS showed reduction in the level of cGMP relative to COCs matured with BSA in the presence of SDF (Fig 2). Previously, negative interference of FCS on cGMP levels had already been observed, and genes controlling nucleotide levels were altered, contributing to decrease cGMP during maturation [17].

In the present study, the inclusion of SDF to elevate cGMP was able to increase nucleotide levels relative to serum, but only when associated with BSA. When associated with serum, cGMP elevation was not sufficient to reach significance, probably due to the high variability in nucleotide quantification in COCs. However, when the lipid content of oocytes was observed in the same treatments, an inverse profile was observed between cGMP and lipid content between groups. That is, the serum group had the lowest level of cGMP and the highest lipid level (Fig 3). The group with the highest level of cGMP (BSA with SDF) had lower lipid content. In addition, the association of SDF to serum reduced lipid content, although the elevated cGMP observed in that group was not sufficient for statistical significance. Our data point to an inverse relation between cGMP levels and lipid content. When cGMP is elevated, it favors lipolysis, leading to lower amounts of lipids, and when reduced, it reduces lipolysis causing greater accumulation of lipids. The relationship of serum on cGMP pathway, which in turn affects lipid metabolism, is still poorly understood, but has already been reported in a previous study [17] and is corroborated by present findings. Serum contains a variety of undefined factors [50] and the identification of which of them could affect cGMP and its signaling in the control of lipid metabolism is still unknown.

We suggest that cGMP affects the lipid metabolism of bovine COCs in a manner similar to that observed in adipocytes [6]. Cyclic GMP effects appear to be due to the activation of PKG, as when a PKG inhibitor was associated in the serum+SDF matured group, the lipid reduction that had been generated by the presence of SDF was reversed, so lipid content increased and was equal to that of the group matured with FBS. The present study and that of Schwarz [17] are suggestive that this pathway may also be explored to better understand the lipid metabolism of COCs and perhaps to be manipulated to reduce lipid accumulation and improve cryotolerance as in studies using cAMP modulators [14, 51, 52].

As serum and cGMP/PKG pathway seemed to be involved in the control of lipid contents, we then studied their effects on transcripts involved in lipolytic activities (*ATGL* and *PLIN2*) in cumulus cells and oocytes. Although transcripts were detected in both cell types, transcript abundance was only affected in cumulus cells, indicating that they would be more sensitive to the effects of serum and/or cGMP on the expression of genes related to the lipid metabolism of COCs (Fig 4). In these cells *ATGL* expression was not altered by serum as previously observed [17], but was greatly reduced by the presence of SDF and this effect was reversed when PKG was inhibited, suggesting a negative control of the expression of this gene by cGMP mediated by PKG. In this case, there appears to be no effect of serum, but the cGMP/PKG pathway may affect the initial process of lipolysis in cumulus cells. The factors that regulate the expression of ATGL remain uncertain even in other cell types [24, 53]. However, regarding the control of its activity, PKA is known to act on specific sites of phosphorylation, which differentiate between species [54, 55], but in cattle this regulation has also never been studied.

PLIN2 is an important regulator of lipolysis, but without enzymatic activities, it is related to the storage of intracellular lipids [28]. This gene was highly expressed in the cumulus cells of COCs matured in medium enriched with FCS showing that, unlike what was observed with *ATGL*, serum can influence its expression. Effects of serum on expression have already been described for other genes, such as related to apoptosis, oxidative stress [56], embryo quality, gap junctions [57] and also including those that control cGMP levels [17]. The increase in *PLIN2* transcripts caused by serum had already been reported in cumulus cells [17, 22, 58] and bovine embryos [32]. These reports and the present study reflect a metabolic response to the enriched media commonly used in *in vitro* cell culture systems, favoring the accumulation of lipids. When the PDE5 inhibitor was associated with serum during IVM, there was a decrease in *PLIN2* expression, and this effect was reversed by the PKG inhibitor. Therefore, *PLIN2* transcription is influenced by serum and cGMP levels and this effect occurs through PKG activity. Thus, accumulation or storage of lipids can be positively influenced when cGMP levels are reduced, as in the presence of serum, or negatively when cGMP levels are elevated, and nucleotide levels may be altered by the presence of serum in culture medium.

According to our results, HSL^ser563^ was detected in bovine oocytes originating from COCs matured in the presence of modulators of the cGMP pathway (FCS, SDF and FCS+SDF+KT), indicating lipolytic activity during maturation. Our data suggest that modulators of the cGMP pathway do not inhibit the HSL^ser563^as it was detected under conditions of high cGMP levels (SDF treatment) and PKG inhibition (FCS+SDF+KT treatment). However, a quantitative analysis would be necessary to determine if there is any variation in HSL activation caused by the cGMP/PKG pathway. Nevertheless, the amount of active HSL enzyme has been described to increase under the influence of PKG [3], besides the classical pathway by PKA in adipocytes [59]. HSL^ser563^ had already been studied in bovine COCs, with detection in oocytes only after maturation [60], in agreement with our observations. In this study [60], although lipid levels were lower in oocytes matured without cumulus cells compared to oocytes matured as COCs, differences relative to the amount of active HSL were not detected between both types of oocytes. Therefore, it is possible that analyzing only levels of active HSL may not be sufficient to show its participation in lipolysis control. In fact, HSL activity depends not only on its phosphorylation, but also on its interaction with other lipid metabolism proteins and translocation from the cytoplasm to the surface of lipid droplets [61], so the intracellular localization of this protein would be an interesting parameter in future studies.

The lack of influence of serum on the main lipolysis catalyzing enzymes, *ATGL* and HSL, suggests that there is no interference on the enzymatic pathway of lipolysis, at least regarding these enzymes. However, its effect on *PLIN2* may be indicative of its interference on the balance of lipid stores, affecting the process of lipid homeostasis. Similar effect in bovine embryos was reported by Al Darwich [62], who supplemented culture medium with polyunsaturated fatty acids (PUFA) which generated a modification of the saturated and unsaturated fatty acid balance in bovine blastocysts, which interfered with the expression of proteins involved in lipid metabolism.

Considering that serum increased lipid contents in oocytes while the association of PDE5 inhibitor decreased them, consequences of such treatments were also evaluated on embryonic development and lipid content (Table 3). PDE5 inhibition during embryo IVC was also analyzed. Under the conditions tested, no influence of PDE5 inhibition was detected on embryo cleavage or on the production of blastocysts (Table 2), which was similar to untreated controls, irrespective of the culture period in which the PDE inhibitor was added (IVM and/or IVC), showing that the inhibitor can be used without impairment to embryonic development.

Regarding the lipid content in embryos, PDE5 inhibition only during maturation caused an increase in lipids, possibly by a compensatory mechanism, as already reported by Schwarz [17]. Embryos from oocytes with lipid reduction, when exposed to the IVC medium containing reduced level of serum during the seven days of *in vitro* development, accumulated more lipids than untreated controls or when PDE5 was inhibited only during IVC. However, when PDE5 inhibitor was added during IVM and also IVC, there was a reduction in embryo lipid content. These observations reinforce the hypothesis of activation of a compensatory mechanism during IVC, which was suppressed by PDE5 inhibition during IVC, possibly avoiding a reduction in cGMP caused by serum during this period. Taken together, results indicate the importance of this pathway for lipolysis.

Strategies to reduce lipid content in oocytes for the production of more cryotolerant embryos have been the target of much research [19, 21, 63–68]. In the present study, blastocysts produced under conditions of PDE5 inhibition during IVM, IVC or both were vitrified, warmed and then cultured for re-expansion and hatching to assess cryotolerance (Table 4). Although there was a reduction of lipids in the embryos in which PDE5 was inhibited during IVM and IVC, this did not translate into better cryotolerance, as re-expansion and hatching rates were similar to untreated controls.

Improvements to cryosurvival after vitrification were observed with the use of inhibitors of fatty acid synthesis [19, 21, 63], lipolytic agents [64, 65], antioxidants [66] or molecules capable of modulating the molecular mechanisms of lipid uptake [67, 68]. A possible explanation for our absence of positive results on cryopreservation in relation to embryos produced with lower lipid content, may be related with other factors that influence their quality and consequently, affecting embryo sensitivity to vitrification [16].

The lipid composition of membranes is crucial under cryopreservation conditions because it affects important physical and chemical properties, particularly membrane fluidity, permeability, and thermal phase behavior [69]. The changes in the lipid structural composition of the membranes, which are not possible to detect using staining procedures, are readily detected by matrix-assisted laser desorption ionization mass spectrometry (MALDI-MS) [70], and the understanding of lipid membranes composition is fundamental to address the difficulties of post cryopreservation embryo survival. Additionally, the oocyte quality [71], season of the year [72] culture conditions [46, 52, 73], cryopreservation method [74], as well as interactions between these factors [75], may affect embryo cryotolerance.

In conclusion, cGMP-PKG pathway participates in the control of lipolysis in oocytes (inverse relationship between cGMP levels and lipid accumulation) and possibly also in cumulus cells (effects on expression of lipolysis-related genes) and embryos (reduced lipid contents by inhibiting cGMP hydrolysis during both IVM and IVC). Serum present in culture media negatively affects this pathway contributing for lipid accumulation, apparently by disturbing cGMP levels, but also by altering lipid droplet associated proteins that control access of lipases to the lipid droplets. Lipases appear not to be affected, but assessment of their activity and intracellular localization, and not only their mRNA expression, would be necessary to confirm this possibility. Inhibiting cGMP hydrolysis contributes, at least in part, to reverse negative effects of serum on cGMP levels and lipolysis, decreasing lipid contents in oocytes and embryos, but without improving embryo cryotolerance. Although with limitations, the present study has contributed with new knowledge regarding lipolysis in bovine COCs and embryos. Further studies are necessary to better understand the complex controls of lipid metabolism in these cells, to allow the development of culture conditions more appropriate to generate *in vitro* produced embryos of better quality and improved cryotolerance.
