# Correction: The role of cGMP as a mediator of lipolysis in bovine oocytes and its effects on embryo development and cryopreservation

**DOI:** 10.1371/journal.pone.0196268

**Published:** 2018-04-18

**Authors:** Kátia R. L. Schwarz, Fernanda C. de Castro, Letícia Schefer, Ramon C. Botigelli, Daniela M. Paschoal, Hugo Fernandes, Cláudia L. V. Leal

The following information is missing from the funding section: This study was supported by CNPq, Brazil, grant # 308216/2014-8 to CLVL.
